# The same domain of Su(Hw) is required for enhancer blocking and direct promoter repression

**DOI:** 10.1038/s41598-019-41761-6

**Published:** 2019-03-29

**Authors:** Larisa Melnikova, Pavel Elizar’ev, Maksim Erokhin, Varvara Molodina, Darya Chetverina, Margarita Kostyuchenko, Pavel Georgiev, Anton Golovnin

**Affiliations:** 1 Department of Drosophila Molecular Genetics, Institute of Gene Biology, Russian Academy of Sciences, 34/5 Vavilov St., 119334 Moscow, Russia; 2 Department of the Control of Genetic Processes, Institute of Gene Biology, Russian Academy of Sciences, 34/5 Vavilov St., 119334 Moscow, Russia

## Abstract

Suppressor of Hairy-wing [Su(Hw)] is a DNA-binding architectural protein that participates in the organization of insulators and repression of promoters in *Drosophila*. This protein contains acidic regions at both ends and a central cluster of 12 zinc finger domains, some of which are involved in the specific recognition of the binding site. One of the well-described *in vivo* function of Su(Hw) is the repression of transcription of neuronal genes in oocytes. Here, we have found that the same Su(Hw) C-terminal region (aa 720–892) is required for insulation as well as for promoter repression. The best characterized partners of Su(Hw), CP190 and Mod(mdg4)-67.2, are not involved in the repression of neuronal genes. Taken together, these results suggest that an unknown protein or protein complex binds to the C-terminal region of Su(Hw) and is responsible for the direct repression activity of Su(Hw).

## Introduction

High-resolution chromosome conformation capture techniques have provided evidence that regulatory elements form loops that are essential for gene regulation in higher eukaryotes^1–6^. In particular enhancers can activate target promoters at large distances (up to hundreds of kb in some cases), which raises the question of the mechanisms regulating such long-distance enhancer–promoter interactions. More than 25 years ago, a special class of regulatory elements, named insulators, was suggested to delimit the activity of enhancers^7–12^. Insulators are defined as regulatory elements that disrupt the communication between an enhancer and a promoter when inserted between them. Some insulator complexes contribute to higher-order organization of chromatin in topologically associated domains that are fundamental elements of the eukaryotic genomic structure^13,14^.

One of the first insulators was identified in the *gypsy* retrotransposon, whose integration into genes often resulted in inactivation of enhancers that were separated from promoters by the *gypsy* insertion^15–19^. The phenotypes of the *gypsy*-induced mutations were suppressed by inactivation of the gene encoding the Suppressor of hairy wing protein [Su(Hw)]^20^. The *gypsy* insulator consists of 12 reiterated binding sites for Su(Hw)^21,22^. Today, Su(Hw) is one of the best characterized insulator proteins. It has been shown that artificial reiterated binding sites for Su(Hw) or *gypsy* insulator can block various enhancers at all stages of *Drosophila* development^21,23–27^.

The Su(Hw) protein contains the N-terminal region involved in the interaction with CP190, an array of 12 C_2_H_2_-type zinc finger domains, and the C-terminal region (aa 716–892) responsible for enhancer blocking activity^28–32^. Several Su(Hw) partners were identified, including Mod(mdg4), CP190, ENY2, Shep, Rump, and HIPP1^28,33–38^. Mod(mdg4)-67.2 is one of the isoforms encoded by the *mod(mdg4)* locus^39,40^. The Mod(mdg4)-67.2 protein contains the N-terminal BTB/POZ domain and glutamine-rich (Q-rich) region which is common to all isoforms and the unique C-terminal region that is required for interaction with the 716–892 aa region of Su(Hw)^28,31,34^. The BTB and Q-rich domains of the Mod(mdg4)-67.2 interact with the M domain of CP190 and the N-terminal region of Su(Hw) (aa 1–238), respectively^31^. The functions of Mod(mdg4)-67.2 are as yet unknown, except for its participation in recruiting the Su(Hw) complex to specific chromatin sites and masking the repression activity of Su(Hw)^31,41,42^.

The CP190 protein contains the N-terminal BTB domain that forms stable homodimers^43–46^. The BTB domain interacts with two adjacent regions at the Su(Hw) N-terminus, which is essential for CP190 recruitment to Su(Hw) sites^32^. CP190 is involved in the recruitment of several transcriptional complexes to chromatin, including the NURF, dREAM, and SAGA complexes^47,48^. CP190 has major effects on chromatin, such as the depletion of nucleosomes and the prevention of heterochromatin expansion^49^. Similar to CP190, ENY2 is recruited to chromatin by interacting with Su(Hw) and participates in the boundary function of Su(Hw), which protects active genes from silencing^36^. Shep and Rump are the RNA binding proteins that function as negative regulators of the Su(Hw) enhancer blocking activity^35,37^. HIPP1 (CG3680) was identified as possible partner of Su(Hw) in a recent screen of proteins interacting with HP1, one of the main heterochromatin proteins^33^. Strong co-localization of HIPP1 and Su(Hw) sites suggests that these proteins are in the same complex^33^.

In previous genome-wide studies, three classes of the Su(Hw) binding regions have been identified, which are characterized by the binding of Su(Hw) alone (SBS-O), both Su(Hw) and CP190 (SBS-C), or three proteins Su(Hw)/CP190/Mod(mdg4)-67.2 (SBS-CM)^50–53^. It has been shown that many SBS-O sites are involved in transcriptional repression^52,54^. The function of Su(Hw) as transcriptional repressor is required for female germline development^54^ and for sustained male fertility^55^.

Here, we made an attempt to identify Su(Hw) domains involved in the repression of several neuronal genes in the ovary. We found that the same C-terminal region (aa 720–892) in Su(Hw) is required for enhancer blocking and for promoter repression.

## Results

### Model system for studying Su(Hw)-dependent repression

**Figure 1:**
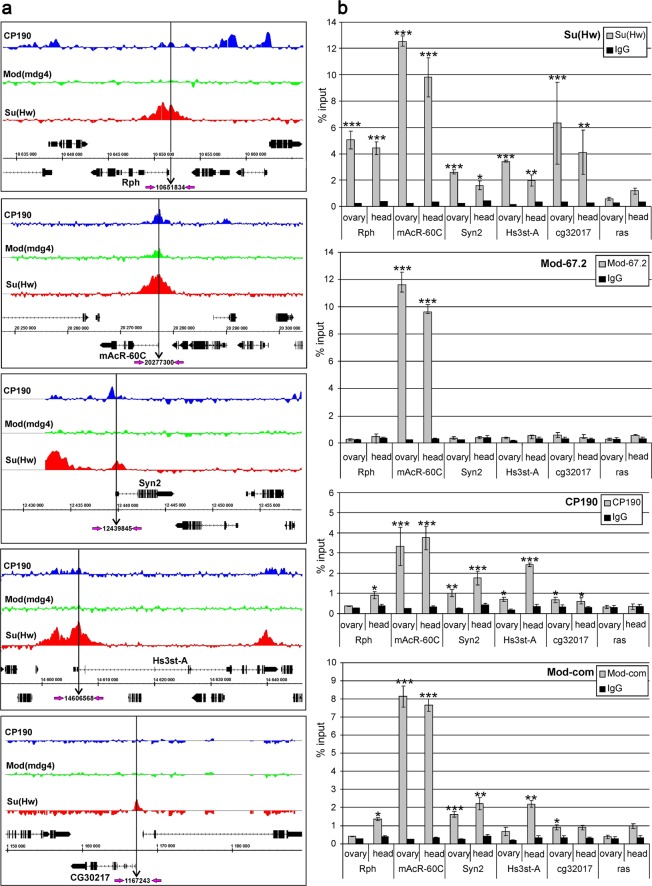
Model system for studying Su(Hw)-dependent repression. (**a**) Genome browser view of insulator protein binding. The regions used in qPCR are indicated by vertical black arrows and numbered according to their chromosome position (FlyBase, 2006). The names of identified genomic regions are given next to the schemes of model genes. (**b**) ChIP-qPCR data on the binding of insulator proteins to the promoters in the ovaries and heads of females. ChIP was performed with antibodies against Su(Hw), Mod(mdg4)-67.2 (Mod-67.2, the C-terminal region corresponding to the specific isoform), CP190, Mod-com (the region common to all Mod(mdg4) isoforms), and nonspecific immunoglobulins (IgG, control). The *ras64B* coding region (ras) was used as a control devoid of Su(Hw) binding sites. The percent recovery of immunoprecipitated DNA (Y axis) was calculated relative to the amount of input DNA. Error bars indicate standard deviation of three independent biological replicates. Statistical analysis (Student’s *t*-test) was performed relative to protein binding to the *ras64B* coding region. Asterisks indicate significance levels of *p < 0.05, **p < 0.01, or ***p < 0.001 (here and in Figs 2–5).

As shown previously, Su(Hw) is involved in the repression of many neural genes in the ovary^54,56^. These genes contain binding sites for Su(Hw) in promoter regions, suggesting the involvement of this protein in direct repression of transcription. To further characterize Su(Hw) domains involved in repression, we selected five representative neural genes whose promoters are bound by Su(Hw) alone (*Rph*, *cg32017*, and *Hs3st-A*), by Su(Hw) and CP190 (*Syn2*), or by Su(Hw), CP190, and Mod(mdg4)-67.2 (*mAcR-60C*) (Fig. 1a). Using ChIP, we confirmed the binding of the insulator proteins to the promoters in the ovaries and heads of females (Fig. 1b). Interestingly, ChIP with a common antibody against all Mod(mdg4) isoforms showed an enrichment of Mod(mdg4) at the promoters. However, Mod(mdg4)-67.2 binds only to the Su(Hw) site at the *mAcR-60C* promoter, suggesting that another Mod(mdg4) isoform was recruited to other promoters by an unknown DNA-bound protein. In general, the observed patterns of Su(Hw), CP190, and Mod(mdg4)-67.2 recruitment to the tested promoters in the ovaries and heads were similar, which is in agreement with the previous observation that these proteins stably bind to their sites in different *Drosophila* tissues and cell lines^51–54^.

**Figure 2:**
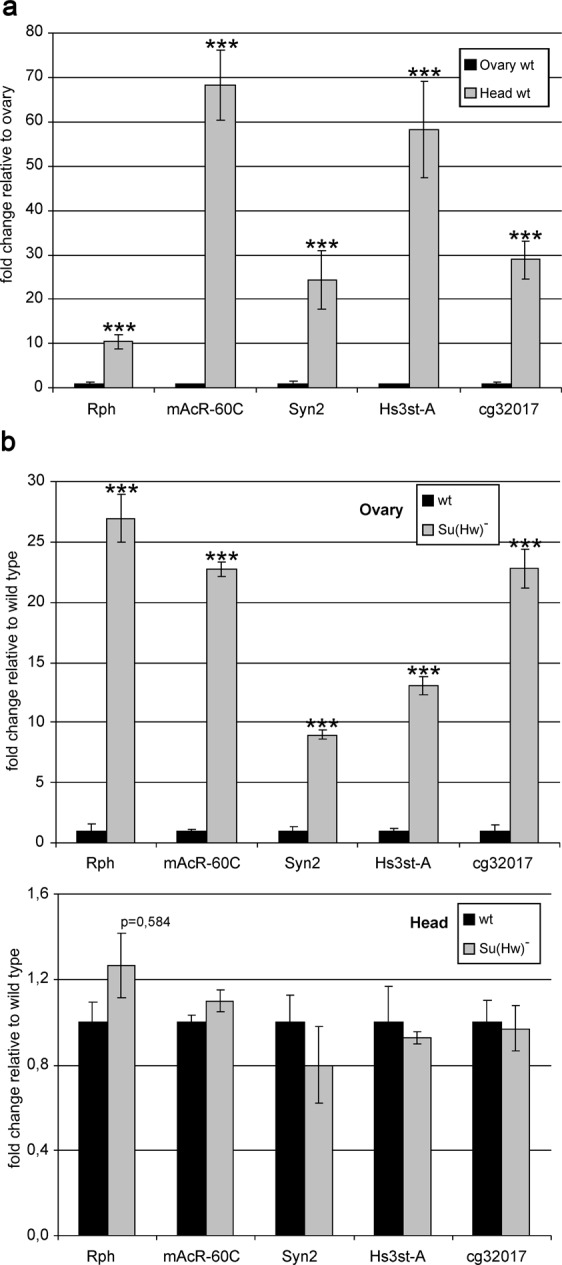
Expression of model genes in the heads and ovaries of females. (**a**) Wild-type (wt) expression of genes, with mRNA levels in the heads shown as a fold change relative to those in the ovaries. (**b**) Expression of genes in the *su(Hw)*^*v*^*/su(Hw)*^*e04061*^ background (su(Hw)^−^), with mRNA levels shown as a fold change relative to the wild-type levels. Error bars indicate standard deviation of three independent biological replicates. Here and in Figs 3–5 and 7, mRNA levels are normalized to that of the *ras64B* gene, which remains unchanged in the *su(Hw)*^−^ background.

We then compared the expression of the model genes in the heads and ovaries of females (Fig. 2a). As expected, the model genes were repressed in the ovaries and actively transcribed in the heads. To confirm the role of Su(Hw) in gene repression, we examined the expression of the model genes in the *su(Hw)*^−^ background. In the absence of the Su(Hw) protein, transcription from the tested genes proved to be increased 10- to 20-fold in the ovaries, while its level in the heads remained unchanged (Fig. 2b). Taken together, these results confirm that the selected genes provide a good model system for the study of Su(Hw)-mediated repression in the ovaries.

### Tissue-specific repression of model genes depends on the presence of Su(Hw) binding sites in promoters

**Figure 3:**
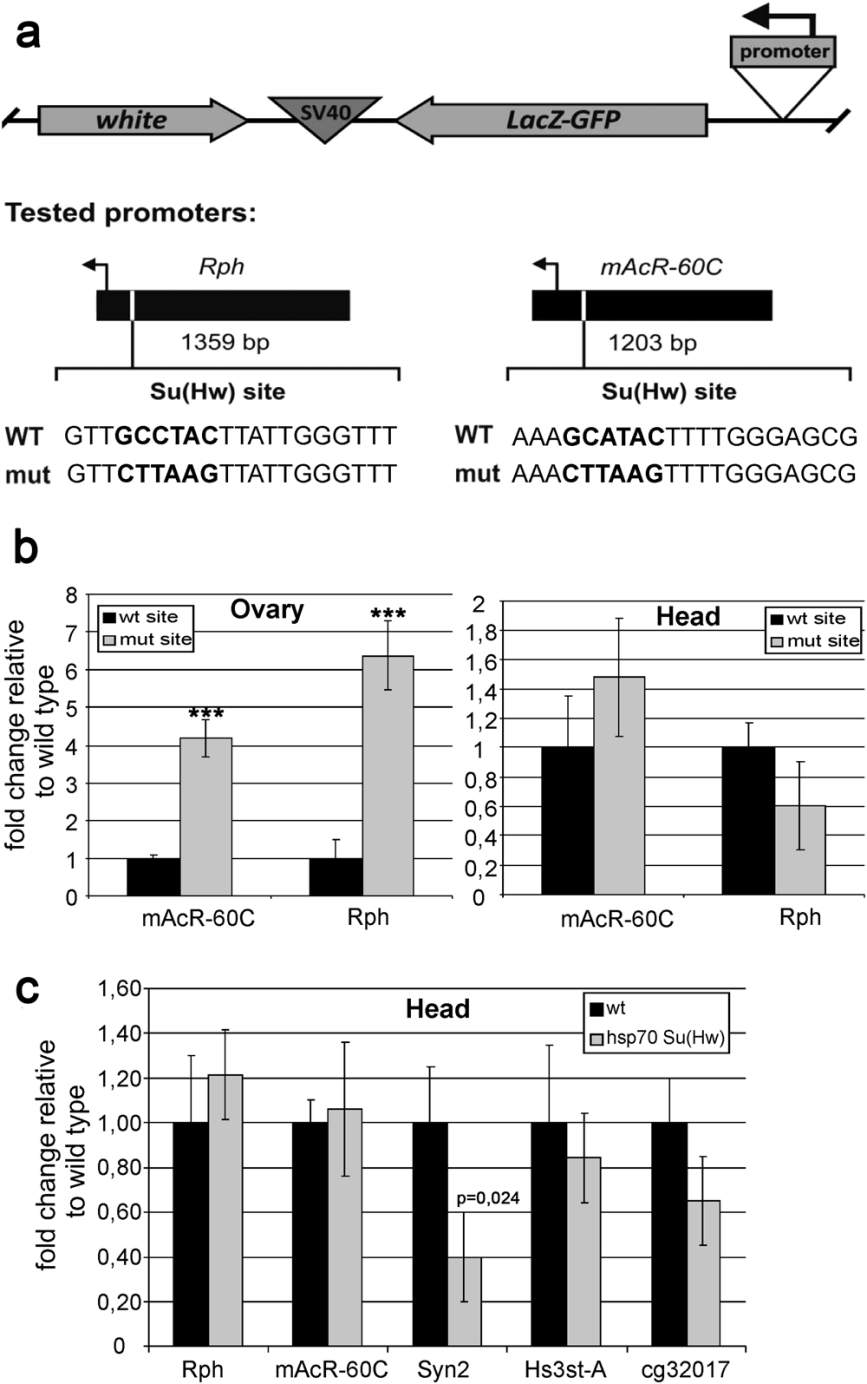
Su(Hw) binding sites are required for repression of transcription. (**a**) Scheme of the constructs (drawn not to scale) containing the *mini white* gene (white), *LacZ-GFP* coding region (LacZ-GFP), transcription terminator from SV40 virus (SV40), and promoters of two model genes, *mAcR-60C* and *Rph* (promoter). Arrowheads show the direction of transcription. The sizes of promoter fragments cloned into the constructs are indicated. WT, wild-type sequences including Su(Hw) binding site (bold type); mut, sequences with mutation in Su(Hw) binding site. (**b**) Expression of two model genes in the wild-type (wt) and mutant (mut) backgrounds. (**c**) Expression of model genes in the heads in the Su(Hw) overexpression background. Expression levels are shown as a fold change relative to wild type. Error bars indicate standard deviation of two independent biological replicates.

There are two possible mechanisms explaining how the Su(Hw) protein can repress the promoters: (1) Su(Hw) competes for the binding site with a transcription factor that activates transcription; (2) Su(Hw) recruits a tissue-specific complex that is responsible for repression. To test these alternative models, we mutated the Su(Hw) binding sites in the promoters of the *mAcR-60C* and *Rph* genes (Fig. 3a). The *lacZ* gene was used as a reporter to test the activity of the promoters, and the *mini-white* gene, which determines eye pigmentation, was used as a marker to select positive transgenic lines. The tested genes were cloned with the *attB* site to allow site-specific integration with the phiC31 integrase system^57^. The constructs were inserted in the same genomic region 38D. The RT-qPCR analysis in the ovaries showed that mutational inactivation of Su(Hw) binding sites led to an increase in transcription, compared to the wild-type level, by a factor of 4 for *mAcR-60C* and by a factor of 6 for *Rph* (Fig. 3b). At the same time, the effect of mutations in the promoters on transcription of the reporter in the heads lacked statistical significance. These results argue against the model that Su(Hw) masks binding site for a transcriptional activator in the promoters.

We also tested whether the overexpression of Su(Hw) can affect transcription of the model genes in the heads. To induce Su(Hw) overexpression, we used the *hsp70* promoter and two-time heat shock treatment for 2 hours at the embryonic and larval stages. The results were confirmed by Western blot analysis (Fig. S1A) and RT-qPCR assay, which revealed approximately 10-fold increase in *su(Hw)* gene transcription, compared to the wild-type level (Fig. S1B). Despite the high level of Su(Hw), we observed a slight repression of only one model gene, *Syn2* (Fig. 3c). This result suggests that, in most cases, the amount of Su(Hw) is not critical for repression. Thus, it is likely that derepression of model genes in the heads is not associated with Su(Hw) displacement from the target promoters.

### The CP190 and Mod(mdg4)-67.2 proteins are not involved in Su(Hw)-mediated repression

**Figure 4:**
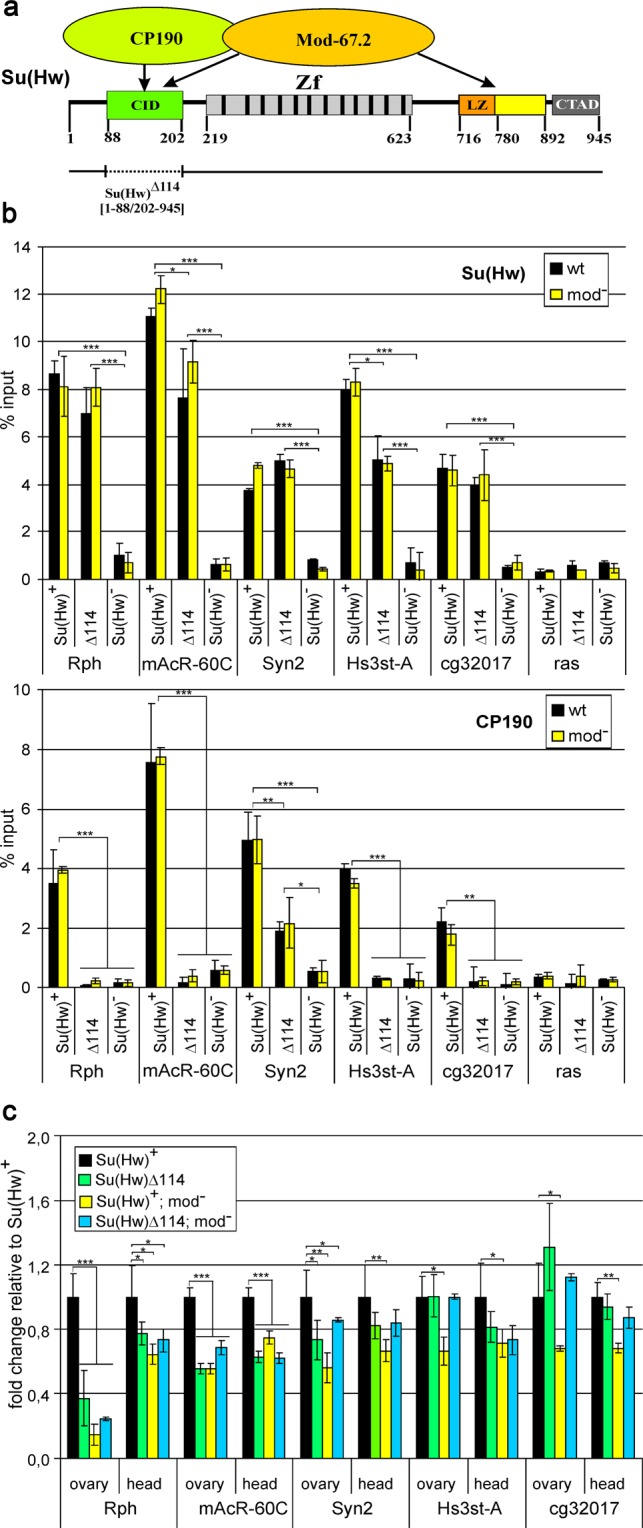
Mod(mdg4)-67.2 and CP190 proteins are not involved in repression. (**a**) Structural scheme of the Su(Hw) protein showing its domains and the corresponding numbers of amino acid residues: CID, CP190 interaction domain; Zf, zinc-finger domain; LZ, leucine zipper motif; CTAD, C-terminal acidic domain. Areas interacting with CP190 and Mod(mdg4)-67.2 proteins are indicated by arrowheads. The horizontal line below shows the Su(Hw) regions contained in the Su(Hw)^Δ114^ derivative, with the dotted fragment indicating the deletion. Numbers in brackets indicate the amino acid residues flanking the protein regions included in the derivative. (**b**) Binding of Su(Hw) and CP190 proteins to the promoters of model genes in pupae of transgenic lines expressing full-length Su(Hw)^+^ and Su(Hw)^Δ114^ in the absence (wt) or presence (mod^−^) of the *mod(mdg4)*^*u1*^*/mod(mdg4)*^*u1*^ mutation. Su(Hw)^−^ refers to the *y*^*2*^ *w*^*1118*^*sc*^*D1*^*ct*^6^; *su(Hw)*^*v*^*/su(Hw)*^*e04061*^ background. The Su(Hw)^Δ114^ derivative is designated Δ114. Different variants of the Su(Hw) protein were expressed in the lines *y*^*2*^ *w*^*1118*^*sc*^*D1*^*ct*^6^; *P{Su(Hw)}-38D/P{Su(Hw)}-38D*; *su(Hw)*^*v*^*/su(Hw)*^*e04061*^, where P{Su(Hw)} refers to Su(Hw)^+^ – *P{w*^+^*;UbqW-Su(Hw)1–945-FLAG};* or Su(Hw)^Δ114^ – *P{w*^+^*;UbqW-Su(Hw)1–88/202–945-FLAG}*. The *ras64B* coding region (ras) was used as a control devoid of Su(Hw) binding sites. The percent recovery of immunoprecipitated DNA (Y axis) was calculated relative to the amount of input DNA. Error bars indicate standard deviation of three independent biological replicates. (**c**) RT-qPCR analysis of model gene expression in the ovaries and heads in the absence or presence (mod^−^) of the *mod(mdg4)*^*u1*^*/mod(mdg4)*^*u1*^ mutation. Expression levels are shown as fold change relative to the Su(Hw)^+^ variant. Error bars indicate standard deviation of three independent biological replicates. Significance leves are shown for treatment comparisons indicated by horizontal brackets.

It has been shown^53,54^ that the CP190 and Mod(mdg4)-67.2 partners of Su(Hw) are not usually essential for repression, since Su(Hw) alone is present at many repressed promoters. As noted above, the *mAcR-60C* promoter is bound by Su(Hw), CP190, and Mod(mdg4)-67.2, while the *Syn2* promoter, only by Su(Hw) and CP190. Hence, the question has arisen whether CP190 and Mod(mdg4)-67.2 are involved in the regulation of these promoters. According to our previous data^32^, the BTB domain of CP190 binds to two regions located between aa 88 and 202 at the N-terminus of Su(Hw) (Fig. 4a). The deletion of these regions in the Su(Hw)^Δ114^ mutants prevents CP190 recruitment to the Su(Hw) complex. We compared the recruitment of Su(Hw) and CP190 proteins to the promoters of model genes in pupae of transgenic lines expressing full-length Su(Hw)^+^ and Su(Hw)^Δ114^ in the absence or presence of the *mod(mdg4)*^*u1*^ mutation inactivating the Mod(mdg4)-67.2 isoform (Fig. 4b). Unexpectedly, no significant effect of the 114-bp deletion on Su(Hw) recruitment was observed even in the absence of Mod(mdg4)-67.2. Thus, Mod(mdg4)-67.2 and CP190 are not required for the Su(Hw) binding to the *mAcR-60C* (Su(Hw)/CP190/Mod(mdg4)-67.2) and *Syn2* (Su(Hw)/CP190) promoters. In the Su(Hw)^Δ114^ mutants, CP190 did not bind to the *mAcR-60C* promoter, confirming the role of Su(Hw) in the recruitment of this protein. In contrast, CP190 still bound to the *Syn2* promoter in these mutants, suggesting that CP190 is recruited to this promoter independently of Su(Hw).

The RT-qPCR analysis in the ovaries and heads showed that the Su(Hw)^Δ114^ mutation did not lead to derepression of model genes (Fig. 4c). In contrast, we observed that in the case of the *Rph* promoter (Su(Hw) alone) and the *mAcR-60C* promoter (Su(Hw)/CP190/Mod(mdg4)-67.2) this mutation resulted in a noticeable decrease of transcription, suggesting the positive role of the corresponding Su(Hw) domain in stimulation of transcription from the promoters. Taken together, these results confirm that the Mod(mdg4)-67.2 and CP190 proteins are not involved in repression.

### The C-terminal domain (aa 720–892) of Su(Hw) is responsible for repression of the promoter

**Figure 5:**
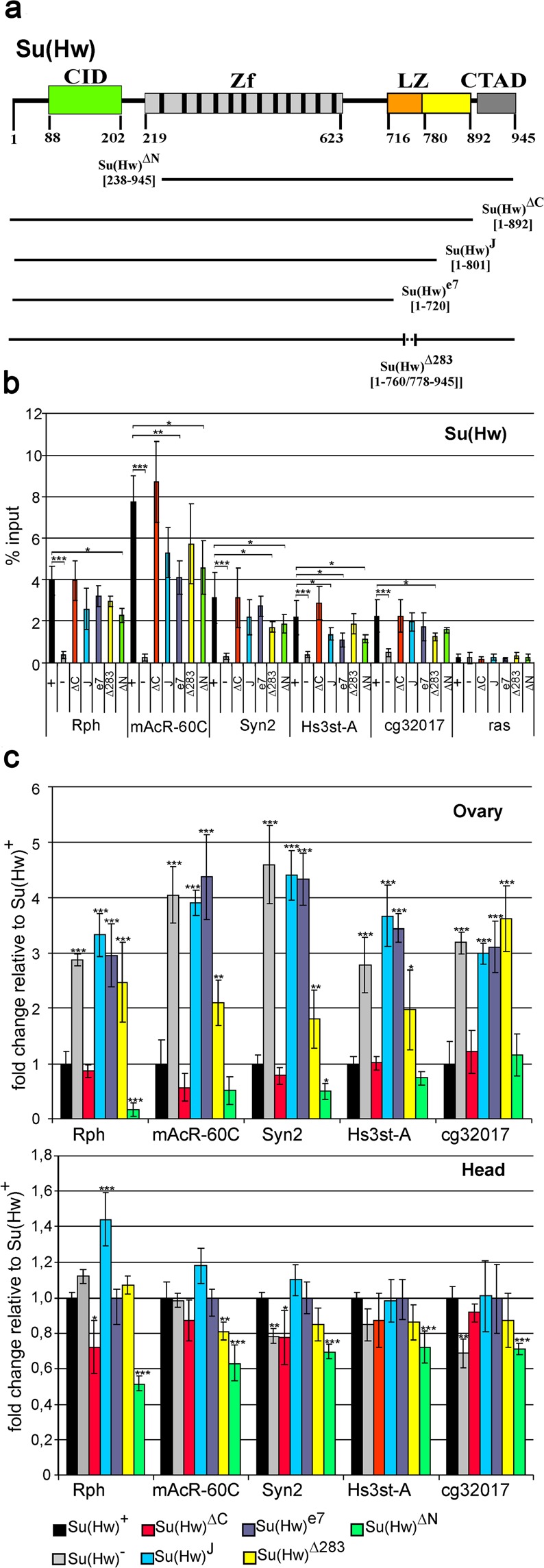
The C-terminal region (720–892 aa) of Su(Hw) is responsible for repression. (**a**) Structural scheme of the Su(Hw) protein, with the lines below representing its derivatives included in analysis. All designations are as in Fig. 4. (**b**) ChIP-qPCR data on Su(Hw) binding to the promoters of model genes in the transgenic lines Su(Hw)^+^ (+), Su(Hw)^ΔC^ (ΔC), Su(Hw)^ΔN^ (ΔN), Su(Hw)^Δ283^ (Δ283), Su(Hw)^e7^ (e7), Su(Hw)^J^ (J), and Su(Hw)^−^ – *y*^*2*^ *w*^*1118*^*sc*^*D1*^*ct*^6^; *su(Hw)*^*v*^*/su(Hw)*^*e04061*^ background (−). Different variants of the Su(Hw) protein were expressed in the lines *y*^*2*^ *w*^*1118*^*sc*^*D1*^*ct*^6^; *P{Su(Hw)}-38D/P{Su(Hw)}-38D*; *su(Hw)*^*v*^*/su(Hw)*^*e04061*^, where P{Su(Hw)} refers to Su(Hw)^+^ – *P{w*^+^*;UbqW-Su(Hw)1–945-FLAG};* Su(Hw)^ΔC^ – *P{w*^+^*;UbqW-Su(Hw)1–892-FLAG};* Su(Hw)^ΔN^ – *P{w*^+^*;UbqW-Su(Hw)238–945-FLAG};* Su(Hw)^Δ283^ – *P{w*^+^*;UbqW-Su(Hw)1–760/778–945-FLAG};* Su(Hw)^e7^ – *P{w*^+^*;UbqW-Su(Hw)1–720-FLAG};* or Su(Hw)^J^ – *P{w*^+^*;UbqW-Su(Hw)1–801-FLAG}*. The *ras64B* coding region (ras) was used as a control devoid of Su(Hw) binding sites. The percent recovery of immunoprecipitated DNA (Y axis) was calculated relative to the amount of input DNA. Error bars indicate standard deviation of three independent biological replicates. Statistical analysis was performed relative to Su(Hw) binding in the Su(Hw)^+^ line. Significance leves are shown for treatment comparisons indicated by horizontal brackets. (**c**) RT-qPCR data on the expression of model genes in the ovaries and heads in the Su(Hw) mutant backgrounds. Expression levels are shown as fold change relative to those in the Su(Hw)^+^ transgenic line. Error bars indicate standard deviation of three independent biological replicates.

Next, we focused on the C-terminal domain of the Su(Hw) protein that contains the acidic and enhancer-blocking regions (aa 892–945 and 716–892, respectively)^28–30^. To map the domains involved in transcriptional repression, we generated transgenes expressing different variants of Su(Hw) under control of the *ubiqitin63-E* promoter (Fig. 5a). The transgenes were inserted in the same 38D genomic region using the phiC31 integrase system^57^. The transgenes were crossed into the *su(Hw)*^−^ (*su(Hw)*^*v*^*/su(Hw)*^*e04061*^ *trans*-heterozygous) background. Western blot analysis showed that all Su(Hw) variants were expressed at similar levels (Fig. S2). The Su(Hw)^ΔC^ line expressed the protein with deletion of the terminal 53 aa, named acidic domain, which was previously shown to be involved in transcriptional repression^32^. The Su(Hw)^Δ283^ line expressed the protein lacking 16 aa (from 760 to 778) that are critical for the enhancer-blocking activity of Su(Hw)^29,30^. In addition, we generated two transgenes mimicking the *su(Hw)*^*J*^ and *su(Hw)*^*e7*^ mutations described previously^29^. The Su(Hw)^e7^ and Su(Hw)^J^ transgenes produced truncated proteins lacking 223 and 150 aa, respectively. Both deletions affect the domain that is essential for interaction with the C-terminal domain of Mod(mdg4)-67.2^28,31,34^. ChIP with pupae showed that all Su(Hw) variants bound to the tested promoters at a level comparable to that of wild-type Su(Hw) (Fig. 5b).

We then compared the effect of Su(Hw) mutants on the expression of model genes in the ovaries and heads (Fig. 5c). In Su(Hw)^J^, Su(Hw)^e7^, or Su(Hw)^Δ283^ females, their expression level in the ovaries proved to be increased up to 5-fold, as in *su(Hw)*^−^ females, while that in the heads expectedly remained unchanged. In contrast, deletion of the C-terminal acidic domain had no effect on the repression activity of the mutant Su(Hw) protein. The expression of different Su(Hw) variants directly correlated with female fertility: Su(Hw)^J^, Su(Hw)^e7^, and Su(Hw)^Δ283^ females were sterile, while Su(Hw)^ΔC^ females were fertile. These results show that the enhancer-blocking domain mediates the Su(Hw) dependent repression of the model promoters.

## Discussion

We have mapped the 720–892 aa region of the Su(Hw) protein, which is responsible for tissue-specific repression of neural genes in the ovaries. This region overlaps with the Su(Hw) enhancer-blocking domain (aa 716–892) characterized previously. Our results show that Su(Hw) overexpression does not lead to increased repression of promoters in the head, suggesting that Su(Hw) itself is not involved in this process. The same deletions in Su(Hw) affect insulation, transcriptional repression and lead to female sterility. As found previously, one copy of the *gypsy* insulator fails to insulate the eye enhancer in the transgenic lines^25^. The Su(Hw) protein in the head fails to function either as an insulator or as a repressor. This provides a basis for the hypothesis that the same unknown complex bound to the 720–892 aa region of Su(Hw) is responsible for the promoter repression and enhancer blocking. It seems likely that this complex is bound to Su(Hw) in many tissues, with the exception of cells attributed to the nervous system. This hypothesis is confirmed by the observation that the *gypsy* insulator cannot block communication between the *iab cis*-regulatory domains and *Abd-B* promoter in the CNS^58^. In addition, the RNA-binding protein Shep has been shown to antagonize Su(Hw) activity in the CNS but not in other tissues^35^. It is possible that Shep competes with the hypothetical enhancer-blocking/repression complex for binding to the Su(Hw) protein.

The Mod(mdg4)-67.2 protein is not required for female fertility and is not involved in the Su(Hw)-mediated repression of the promoters^56^. At the same time, Mod(mdg4)-67.2 affects the enhancer-blocking activity of Su(Hw)-dependent insulators^25,34,38,41,59,60^. Mod(mdg4)-67.2 can interact with proteins responsible for long-distance communication between enhancers and promoters. For example, Mod(mdg4)-67.2 interacts with Zeste, which is essential for long-range interactions between the eye enhancer and *white* promoter^25,61^.

Interestingly, Mod(mdg4)-67.2 protein interacts with the 716–892 aa region of Su(Hw) that includes a leucine zipper motif and completely overlaps with the enhancer blocking/repression domain^28,34^. In the *y*^*2*^ model system^62,63^, the Su(Hw) complex acts as an insulator, while in the absence of Mod(mdg4)-67.2 it becomes a repressor^41,64^, suggesting that Mod(mdg4)-67.2 competes with the hypothetical enhancer-blocking/repression complex. This hypothesis is consistent with the observation that the genomic sites that bind the Su(Hw) protein in the absence of Mod (mdg4)-67.2 act as negative regulators of transcription^52,54^.

As shown previously, CP190 mediates protein–protein interactions necessary for the establishment of long-term contacts between insulator complexes^46^. Moreover, CP190 protein is critical for the functioning of the Fub insulator element that topologically separates the *Ubx* gene from the posterior *abd-A* gene^60^. CP190 is also involved in the recruitment of transcriptional complexes that open chromatin and stimulate transcription^49,65^. We have demonstrated that deletion of the Su(Hw) N-terminal domain responsible for the interaction with CP190 leads to a significant decrease in the expression of model genes, suggesting that CP190 is involved in transcription activation rather than in Su(Hw)-dependent repression.

The CP190 and Mod(mdg4)-67.2 proteins facilitate the binding of Su(Hw) to many SBS-CM sites and, in particular, to the *gypsy* insulator^31,42^. Su(Hw) binds to the *mAcR-60C* promoter in complex with CP190 and Mod(mdg4)-67.2. However, deletion of the Su(Hw) N-terminal domain even in combination with the *mod(mdg4)*^*u1*^ mutation has no effect either on Su(Hw) binding or on promoter repression. Thus, Su(Hw) recruitment to the *mAcR-60C* promoter does not depend on the presence of Mod(mdg4)-67.2 or CP190. It is noteworthy in this context that CP190 is recruited to the *Syn* promoter independently of Su(Hw). Our previous data show that multiple interactions between CP190, Mod(mdg4)-67.2, and Su(Hw) are involved in the formation of a stable DNA-bound chromatin complex^31,32^. Based on this fact, we hypothesize that only the Su(Hw)/Mod(mdg4)-67.2/CP190 complex or Su(Hw) in combination with as yet unknown proteins are bound to chromatin, while SBS-C sites are formed by independent binding of the Su(Hw) protein and CP190 in complex with another architectural protein.

It was previously speculated that Su(Hw) repression might depend on the recruitment of the LINT complex^54^. This transcriptional repressor complex of *Drosophila* contains three subunits: CoREST, lethal (3) malignant brain tumor protein (L(3)mbt), and L(3)mbt interacting protein 1 (dLint-1)^66,67^. As shown previously, over a third of CNS-enriched repressed target genes contain SBSs that colocalize with L(3)mbt and half of them contain SBSs that colocalize with dLint-1^54^. Based on this finding, we examined whether L(3)mbt colocalized with the promoters of our model genes (Fig. S3). We analyzed L(3)mbt occupancy in the larval CNS and in Kc cells (female embryonic cell line) in comparison with Su(Hw) and CP190 localization^53,67,68^. We did not observe precise tissue specific occupancy of L(3)mbt on the tested promoters: this proteint was either enriched in the larval CNS and Kc cells (Rph) or absent in both cases (Hs3st-A, Syn2, and mAcR-60). It was only on the cg32017 promoter that L(3)mbt was enriched in Kc cells and not detected in the CNS. Apparently, L(3)mbt is not directly recruited to SBSs by Su(Hw). This assumption is confirmed by results of yeast two hybrid assay (Suppl. Table 1), which show that L(3)mbt directly interacted with CP190 but not with Su(Hw). Since CP190 is not directly involved in transcriptional repression, L(3)mbt is unlikely to play a general role in repression of tested genes. A recent screen of proteins interacting with HP1 has identified HIPP1 as a possible partner of Su(Hw)^33^. We have shown that HIPP1 dirrectly interacts with Su(Hw) region responsible for repression (unpublished data). As demonstrated redcently, however, HIPP1 is dispensable for the repression activity of Su(Hw)^69^. Taken together, these results suggest that additional, as yet unidentified complex is responsible for tissue-specific repression of neural genes in the ovaries. Further studies are needed to identify components of this complex and mechanism of its formation on SBSs.

## Materials and Methods

The transgenic constructs are described in the Supplementary Materials.

### Germ-line transformation, genetic crosses, and phenotypic analysis

All flies were maintained at 25 °C on the standard yeast medium. To obtain transgenic flies with insertion in 38D, the DNA of reporter constructs was injected into preblastoderm embryos of *y*^*1*^ M{vas-int.Dm}ZH-2A w*; M{3xP3-RFP.attP′}ZH-38D genotype^57^. The generation of transgenic lines and construct introduction into the *Su(Hw)*^*v*^*/Su(Hw)*^*e04061*^ background were performed as described^41^.

### Dissection of flies

For each replicate, approximately 100 ovaries from 4- to 6-hour-old virgin females were dissected as described previously^51,52^. After dissection, the flies were collected into a 50-mL Falcon tube, frozen in liquid nitrogen, and vigorously vortexes for 1 min. The contents of the tube were poured onto a sheet of white paper, and the heads of flies were separated from the bodies with a bird feather.

### RNA isolation and RT-qPCR analysis

For RT-qPCR experiments, total RNA from the heads and ovaries of females was isolated with TRIzol reagent (Invitrogen). Genomic DNA was removed by treatment with DNase I (Fermentas, 1 U per 10 μg) followed by purification with a QIAGEN RNeasy kit. RNA was reverse transcribed into cDNA with a RevertAid H Minus RT Revert Transcriptase (Fermentas) following the manufacturer’s instructions. The resulting cDNA was analyzed by quantitative PCR (Bio-Rad CFX 96 Cycler) using SYBR Green. Relative steady-state mRNA levels were determined from the threshold cycle for amplification using the ΔΔCT method. Each experiment was performed in two to three independent biological replicates, and the results were averaged. The expression level of each gene was determined using *ras64B* as an internal control. Primer sequences used in real-time PCR analysis are listed in Suppl. Table 2.

### Chromatin preparation and ChIP analysis

Chromatin was prepared either from the heads and ovaries of female flies or from middle pupae. The material (150–200 mg) was homogenized in 5 mL of buffer A (15 mM HEPES, pH 7.6 with 60 mM KCl, 15 mM NaCl, 4 mM MgCl_2_, 0.5% Triton X100, 0.5 mM DTT, 10 mM sodium butyrate, EDTA-free protease inhibitors cocktail, and 1.8% formaldehyde) at room temperature using first a Potter homogenizer and then a Dounce homogenizer with type A pestle (three strokes). The protease inhibitors cocktail (Roche, Cat #1873 580) was used following the manufacturer’s instructions. After 15 minutes (total time starting from beginning of homogenization), glycine solution was added to a concentration of 225 mM, and the mixture was stirred, incubated for 5 minutes, and then centrifuged at 4000 *g* and 4 °C for 5 minutes. The pellet was washed three times with buffer A at 4 °C and resuspended in 0.5 mL of lysis buffer (15 mM HEPES, pH 7.6 with 140 mM NaCl, 1 mM EDTA, 0.5 mM EGTA, 1% Triton X-100, 0.5 mM DTT, 0.1% sodium deoxycholate, 0.1% SDS, 0.5% N-lauroylsarcosine, 10 mM sodium butyrate, and protease inhibitors cocktail). The mixture was incubated on a rotary shaker at 4 °C for 10 minutes, sonicated on ice with a Branson Sonifier 450 (power 2, duty cycle 100%, time 4630 seconds at 2-second intervals), rotated for another 10 minutes, and then centrifuged at 15,000 *g* for 5 minutes. The supernatant was transferred to a new tube. The pellet was resuspended in 0.5 mL of lysis buffer, incubated on a rotary shaker for 10 minutes, and centrifuged again. The first and second supernatants were pooled and centrifuged twice at 15,000 *g* for 10 minutes. The supernatant was diluted with ten volumes of ChiP Dilution Buffer (16.7 mM Tris-HCl, pH 8.0 with 0.01% SDS, 1.1% Triton X-100, 1.2 mM EDTA, and 167 mM NaCl) and, to reduce nonspecific background, pre-cleared by incubation with protein A or protein G agarose beads for 30 min at 4 °C, with constant stirring. Agarose was pelleted by brief centrifugation, and the supernatant was collected for chromatin immunoprecipitation with appropriate antibodies (see below). After overnight incubation at 4 °C on a rotary shaker, protein A or protein G agarose beads were added to collect the precipitated complexes, and incubation was continued for 2 hours under the same conditions. Agarose was pelleted by centrifugation (700–1000 rpm at 4 °C, ~1 minute), the supernatant was carefully removed, and the pellet was washed with the following buffers (1 mL each, for 3–5 minutes on a rotary shaker): Low Salt Wash Buffer (20 mM Tris-HCl, pH 8.0 with 0.1% SDS, 1% Triton X-100, 2 mM EDTA, and 150 mM NaCl), High Salt Wash Buffer (20 mM Tris-HCl, pH 8.0, with 0.1% SDS, 1% Triton X-100, 2 mM EDTA, and 500 mM NaCl), LiCl Wash Buffer (10 mM Tris-HCl, pH 8.0, with 0.25 M LiCl, 1% NP40, 1% deoxycholate, and 1 mM EDTA,), and two portions of TE Buffer. The complex was removed from the agarose by two rounds of incubation with 250 μL of elution buffer (1%SDS, 0.1 M NaHCO_3_) at room temperature for 15 minutes, with rotation. The eluates were pooled, supplemented with 20 μL of 5 M NaCl, and heated at 65 °C for 4 hours to reverse the complex–DNA crosslinks. Then 20 μL of 1 M Tris-HCl (pH 6.5), 10 μL of 0.5 M EDTA, and 2 μL of Proteinase K solution (10 mg/mL) were added, and the mixture was incubated at 45 °C for 1 hour. DNA was recovered by phenol/chloroform extraction and ethanol precipitation and solubilized in water for PCR. PCR products were amplified from at least two separate immunoprecipitates from three different chromatin preparations. Primer sequences used in PCR for ChIP analysis are shown in Suppl. Table 3.

### Antibodies

Specific antibodies and working dilutions were as follows: rat anti-CP190 (1:500), rabbit anti**-**Mod(mdg4)-67.2 (1:500), mouse anti-Mod-common (1:500), and rabbit anti Su(Hw) N-terminal domain (1:200) raised in our lab^42,70^; rabbit antibodies against the C-terminal domain of Su(Hw) (1:200) kindly provided by M. Erokhin^32^.

## Supplementary information

Dataset 1

## Data Availability

All data generated or analyzed during this study are included in this published article and its Supplementary Information file.
