# Supplementary material for: The same domain of Su(Hw) is required for enhancer blocking and direct promoter repression
Source: Sci Rep. 2019 Mar 29;9:5314. doi: 10.1038/s41598-019-41761-6 (PMC6441048; doi:10.1038/s41598-019-41761-6)
Supplement: Supplementary file 1 — Dataset 1 [file 41598_2019_41761_MOESM1_ESM.pdf]

# The same domain of Su(Hw) is required for enhancer blocking and direct promoter repression

Larisa Melnikova[a], Pavel Elizar'ev [b], Maksim Erokhin[b], Varvara Molodina[a], Darya Chetverina[b], Margarita Kostyuchenko[a], Pavel Georgiev[b,]* and Anton Golovnin[a,]*

[a]*Department of Drosophila Molecular Genetics, Institute of Gene Biology, Russian Academy of Sciences, 34/5 Vavilov St., 119334 Moscow, Russia*

[b]*Department of the Control of Genetic Processes, Institute of Gene Biology, Russian Academy of Sciences, 34/5 Vavilov St., 119334 Moscow, Russia*

**Corresponding authors:*

Anton Golovnin[1]. E-mail: agolovnin@mail.ru

Pavel Georgiev[2]. E-mail: georgiev_p@mail.ru

## Supplementary Figures

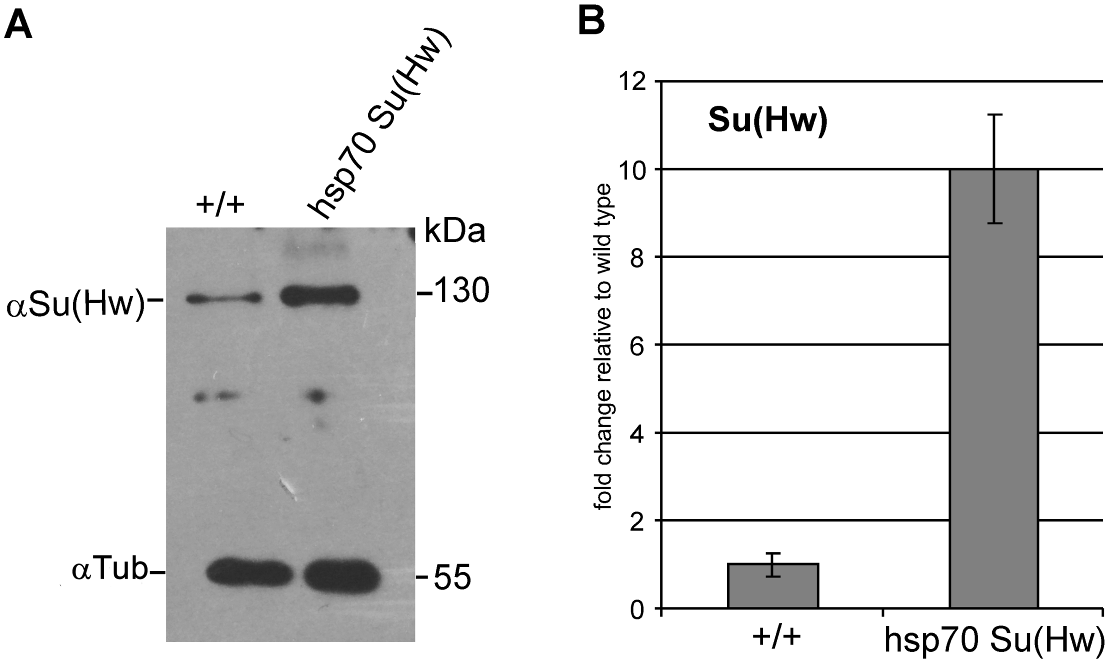


**Suppl. Figure 1. Overexpression of Su(Hw) protein.**

(A) Su(Hw) isolated from adult flies as described [1] was resolved by electrophoresis in 7.5% SDS-PAAG, electroblotted onto a PVDF membrane, and simultaneously probed with antibodies against Su(Hw) (designated αSu(Hw)) and against Tub-1A2 (Abcam #11325) (designated αTub). Anti-tubulin staining (αTub) was used as loading control. Lines included in analysis are indicated above the figure: +/+ – $y^2\ w^{1118} sc^{D1} ct^6$; hsp70 Su(Hw) – $y^2\ w^{1118} sc^{D1} ct^6$; *P{*$w^+$*hsp70-Su(Hw)}/ P{*$w^+$*hsp70-Su(Hw)}*.

To induce Su(Hw) overexpression by the *hsp70* promoter, we used two-time heat shock treatment for 2 hours at the embryonic and larval stages.

(B) Expression of *su(Hw)* gene in the wild type (+/+) and under *hsp70* driver (hsp70 Su(Hw)), with mRNA levels normalized to the *ras64B* gene and shown as a fold change relative to wild type. Error bars indicate standard deviation of two independent biological replicates.

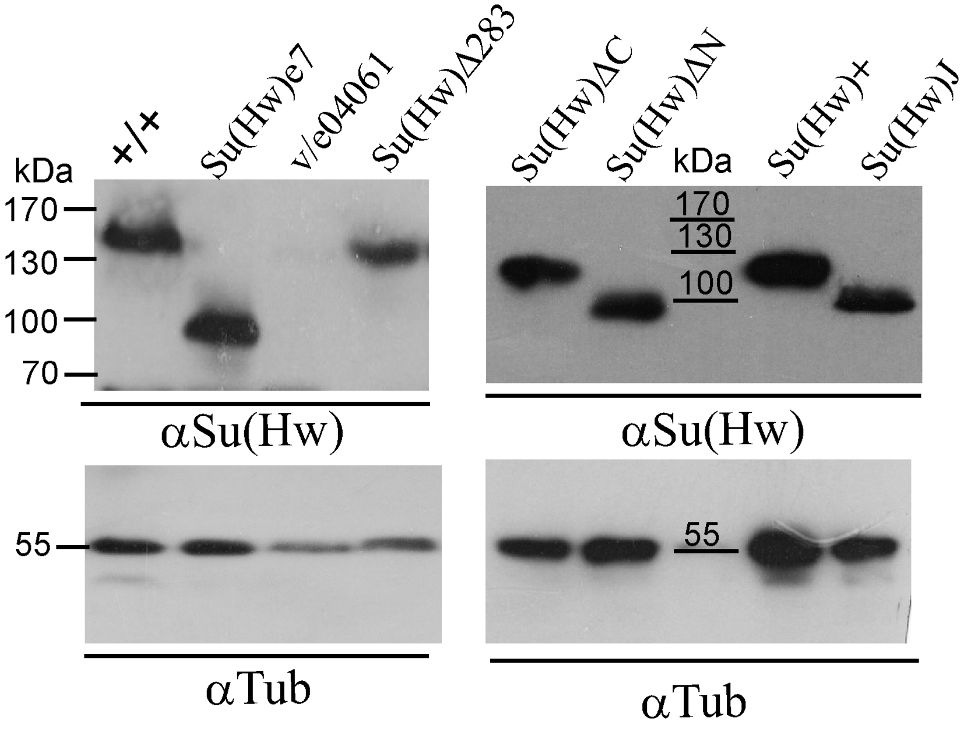


**Suppl. Figure. 2. Western blot analysis of the expression of Su(Hw) protein and its derivatives *in vivo*.**

Su(Hw) isolated from adult flies as described [1] was resolved by electrophoresis in 7.5% SDS-PAAG, and electroblotted onto a PVDF membranes. The PVDF membranes were consecutively probed with antibodies against Su(Hw) (designated αSu(Hw)) and against Tub-1A2 (Abcam #11325) (designated αTub). Anti-tubulin staining (αTub) was used as loading control. Lines included in analysis are indicated above the figure. The images are cropped.

Expression analysis of transgenic lines was performed on the $y^{2}$ $w^{1118}sc^{D1}ct^{6}$; $su(Hw)^{v}/su(Hw)^{e04061}$ background designated as v/e04061; +/+ is the $y^{2}$ $w^{1118}sc^{D1}ct^{6}$ line. Different variants of the Su(Hw) protein were expressed in the lines $y^{2}$ $w^{1118}sc^{D1}ct^{6}$; *P{Su(Hw)}-38D/P{Su(Hw)}-38D*; $su(Hw)^{v}/su(Hw)^{e04061}$, where P{Su(Hw)} refers to

$Su(Hw)^{+}$ – *P{*$w^{+}$*;UbqW-Su(Hw)1-945-FLAG}/ P{*$w^{+}$*;UbqW-Su(Hw)1-945-FLAG};*

$Su(Hw)^{\Delta C}$ – *P{*$w^{+}$*;UbqW-Su(Hw)1-892-FLAG}/ P{*$w^{+}$*;UbqW- Su(Hw)1-892-FLAG};*

$Su(Hw)^{\Delta N}$ – *P{*$w^{+}$*;UbqW-Su(Hw)238-945-FLAG}/ P{*$w^{+}$*;UbqW- Su(Hw)238-945-FLAG};*

$Su(Hw)^{\Delta 283}$ – *P{*$w^{+}$*;UbqW-Su(Hw)1-760/778-945-FLAG}/ P{*$w^{+}$*;UbqW- Su(Hw) 1-760/778-945-FLAG};*

$Su(Hw)^{e7}$ – *P{*$w^{+}$*;UbqW-Su(Hw)1-720-FLAG}/ P{*$w^{+}$*;UbqW- Su(Hw)1-720-FLAG};* or

$Su(Hw)^{J}$ – *P{*$w^{+}$*;UbqW-Su(Hw)1-801-FLAG}/ P{*$w^{+}$*;UbqW- Su(Hw)1-801-FLAG}.*

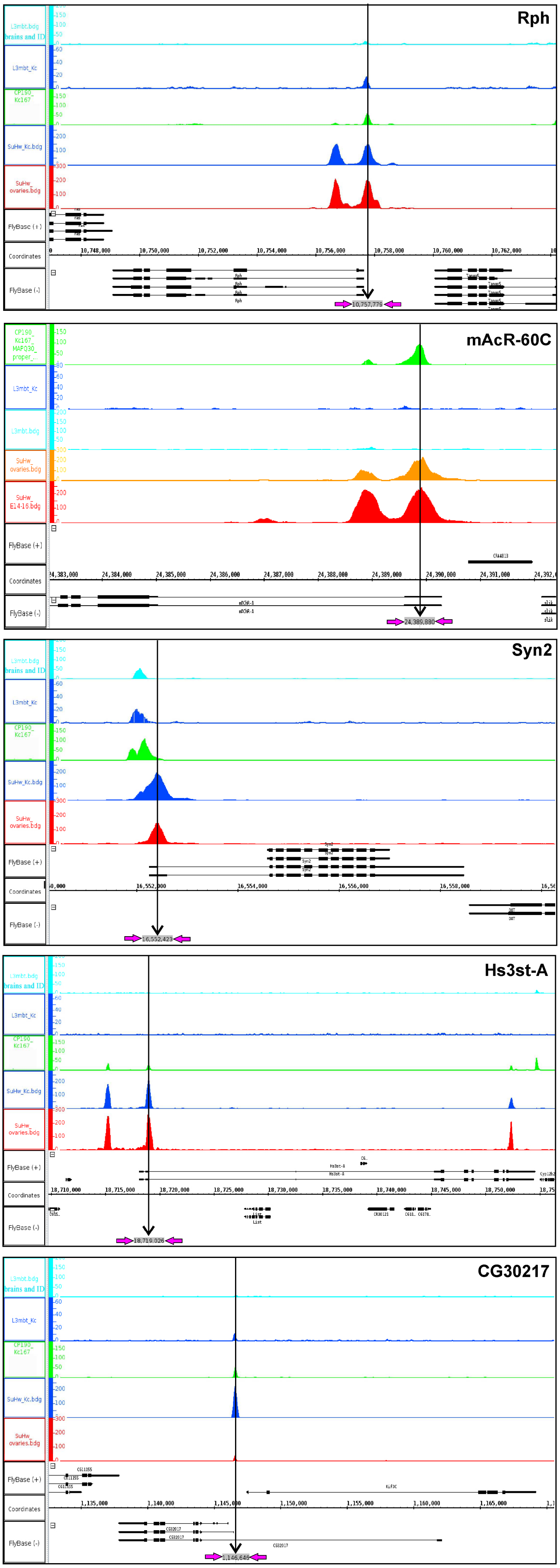


**Suppl. Figure. 3. The occupancy of L(3)mbt protein on tested promoters.** FASTQ files from corresponding GEO/Sequence Read Archive database were processed using bowtie 2 software [2,3] to map sequence reads containing fewer than two mismatches to the fly genome (BDGP Release 6). Bowtie2 output files were analyzed with the MACS ver. 2.1.2 with default parameters. Of the peaks given by MACS, only those with fold enrichment above 2 and coverage above 20× were kept. The regions used in qPCR are indicated by vertical black arrows and numbered according to their chromosome position (FlyBase, 2014).

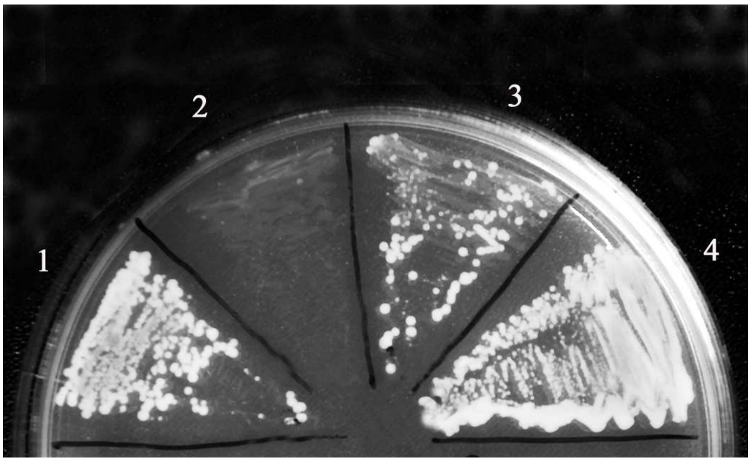


**Suppl. Figure. 4. Representative examples of colony growth in yeast two-hybrid assay.**
(1) Intermediate growth of yeast colonies on selective plate, designated as "++". (2) No colony growth indicating the absence of interaction, designated as "–", which is observed in negative control. (3) Weak growth of yeast colonies on selective plate, designated as "+". (4) Abundant growth of yeast colonies on selective plate, designated as "+++".

# Supplementary Materials

## Transgenic constructs

Constructs **P{w$^+$;UbqW-Su(Hw)1-954-FLAG}** and **P{w$^+$;UbqW-Su(Hw) 238-945-FLAG}** were described previously [4].

Construct **P{w$^+$;UbqW-Su(Hw)1-892-FLAG}** was generated by cleaving the PCR product generated from Su(Hw) cDNA with plasmid primer and 5'-aaagcttttcgcctgtgaccgacac-3'primer. This PCR product was cleaved by *Kpn*I and *Hind*III and cloned into pUbqWb vector cleaved with the same enzymes.

To generate the **P{w$^+$;UbqW-Su(Hw)1-801-FLAG}, P{w$^+$;UbqW-Su(Hw)1-720-FLAG}**, and **P{w$^+$;UbqW-Su(Hw) 1-760/778-945-FLAG}** constructs, the *Kpn*I–*Sac*I fragment (filled in with Klenow fragment) from pAc5.1 vector containing corresponding cDNA of Su(Hw) protein fused to FLAG epitope was cloned into pUbqWB cleaved with *Kpn*I and *Bam*HI (filled in with Klenow fragment).

To generate **P{w$^+$;Pr-LacZ-GFP}**, where Pr is either *Rph* or *mAcR-60C* promoter, the *loxP* sequence was inserted into CΔ plasmid [5] cleaved with *Bam*HI and *Eco*RI [loxP-CΔ]. The attB fragment PCR-amplified with primers 5'-gtcgacgatgtaggtcacgg-3' and 5'-gtcgacatgcccgccgtgac-3' was inserted into loxP-CΔ plasmid to create the attB-loxP-CΔ vector completely analogous to the system used in [6].

The 4.8-kb fragment corresponding to *LacZ-GFP* coding region fusion followed by SV40 terminator was cloned into *Eco*RI site of attB-loxP-CΔ plasmid to create attB-LG-loxP-CΔ. The 1300-bp Rph promoter (5'-agcggccgctgatacacctctccgaggcg- 3' and 5'-tgcggccgcgtttgcgaatcggttgcgaa-3') and 1204-bp mAcR60C (5'-agcggccgcagtcccattgaaaagtggc-3' and 5'-tgcggccgcctcgttctcgctccctctc-3') were PCR-amplified with primers containing *Not*I sites. Both promoters were inserted upstream of the *LacZ-GFP* fusion reporter into the attB-LG-loxP-CΔ vector at *Not*I site. The promoters with mutated Su(Hw) sites were created by site-directed mutagenesis by PCR and inserted at *Not*I site into the attB-LG-loxP-CΔ vector.

## References

1 Gdula, D. A. & Corces, V. G. Characterization of functional domains of the su(Hw) protein that mediate the silencing effect of mod(mdg4) mutations. *Genetics.* **145**, 153-161 (1997).

2 Langmead, B. & Salzberg, S. L. Fast gapped-read alignment with Bowtie 2. *Nat Methods* **9**, 357-359 (2012).

3 Langmead, B., Wilks, C., Antonescu, V. & Charles, R. Scaling read aligners to hundreds of threads on general-purpose processors. *Bioinformatics* **35**, 421-432 (2019).

4 Melnikova, L. *et al.* Multiple interactions are involved in a highly specific association of the Mod(mdg4)-67.2 isoform with the Su(Hw) sites in Drosophila. *Open Biol.***7**, doi:10.1098/rsob.170150 (2017).

5 Chetverina, D. *et al.* Red flag on the white reporter: a versatile insulator abuts the white gene in Drosophila and is omnipresent in mini-white constructs. *Nucleic Acids Res.* **36**, 929-937 (2008).

6 Bischof, J., Maeda, R. K., Hediger, M., Karch, F. & Basler, K. An optimized transgenesis system for Drosophila using germ-line-specific phiC31 integrases. *Proc Natl Acad Sci U S A.* **104**, 3312-3317 (2007).

## Supplementary Table 1.

**Summary of yeast two-hybrid analysis of insulator proteins for interaction with L(3)mbt**

| | **L(3)mbt bait** | **L(3)mbt prey** | **CP190 bait** | **CP190 prey** |
|---|---|---|---|---|
| **Su(Hw)** | – | – | +++ | +++ |
| **Mod(mdg4)-67.2** | – | – | +++ | +++ |
| **CP190** | ++ | ++ | +++ | +++ |
| **pGBT9/pGAD424** | – | – | – | – |

Two-hybrid assays were carried out using yeast strain pJ694A, plasmids, and protocols from Clontech. For growth assays, plasmids were transformed into yeast strain pJ694A by the lithium acetate method, as recommended by the manufacturer, and plated onto media without tryptophan and leucine. After 2 days of growth at 30°C, the cells were plated onto selective media without tryptophan, leucine, histidine, and adenine, and characteristics of their growth were compared. No growth was observed after transformation with single vectors (pGBT9/pGAD424), indicating that interactions between the proteins were required for the expression of the reporter genes (data not shown). As negative control it was used vector with activating GAL4 domain while tested protein was fused with DNA binding domain of GAL4 to verify the level of self-activation. None of the tested proteins showed self-activation in control experiments. CP190 as bait or prey was used as a positive control. The (+) signs indicate the relative strength of the two-hybrid interaction, and the (–) sign indicates the absence of interaction (see Fig. S4).

## Supplementary Table 2.

### Primer sequences used in RT-qPCR analysis

| Primer pair | Sequences |
|---|---|
| mAcR-60C G d<br>mAcR-60C G r | 5'-CTGCTTGGGTATGACCTTGGCGTTG-3'<br>5'-TTCAGCCTGCCACTCGGACGG-3' |
| Rph G d<br>Rph G r | 5'-AACAACTCCGGCAACTCACAAACG-3'<br>5'-AGGCCTCACGATAGCTAATGGCAA-3' |
| Syn2 G d<br>Syn2 G r | 5'-AATGCCAAGTCCTGCAAAGTCTGCTG-3'<br>5'-TATATCATTTTGAGTGTGAGTCCATGCG-3' |
| Hs3st-A RT d<br>Hs3st-A RT r | 5'-CAAGGGCTTCTACTGTCTG-3'<br>5'-AAATCGCTGATTGTACTCGG-3' |
| cg32017 RT d<br>cg32017 RT r | 5'-CGTATTCCATTTGAACACCATC-3'<br>5'-GCTTCCTTGTATAGCCACTGA-3' |
| Ras fw<br>Ras rev | 5'-GAGGGATTCCTGCTCGTCTTCG-3'<br>5'-GTCGCACTTGTTACCCACCATC-3' |

## Supplementary Table 3.

### Primer sequences used in ChIP-qPCR analysis

| Primer pair | Sequences |
|---|---|
| mAcR-60C TS2 d<br>mAcR-60C TS2 r | 5'-CGCTCTCTCTCGGCTGCT-3'<br>5'-CGCGTTTGTTTTTCACTCGGTTA-3' |
| Rph TS d<br>Rph TS r | 5'-TCCCTACCGGCTGCGATAAC-3'<br>5'-GCTGCGGAGCGTTTGCGAAT-3' |
| Syn2 TS d<br>Syn2 TS r | 5'-ACTGCGTGCCGCTTTTGCT-3'<br>5'-AAACTGGCTGGCTGTACTCC-3' |
| Hs3st-A Su d<br>Hs3st-A Su r | 5'-AGCACGAAAAGTGGCTACAA-3'<br>5'-CTCGTTATGGGTTAAAGCTGTT-3' |
| cg32017 TS d<br>cg32017 TS r | 5'-TGACCACTGTAACCTGAAAAGA-3'<br>5'-GTACCGTGTACCTGTAATGAAT-3' |
| Ras fw<br>Ras rev | 5'-GAGGGATTCCTGCTCGTCTTCG-3'<br>5'-GTCGCACTTGTTACCCACCATC-3' |
